# Healthcare bias and health inequalities towards displaced Syrians in Lebanon: a qualitative study

**DOI:** 10.3389/fpubh.2023.1273916

**Published:** 2023-11-30

**Authors:** Riwa Khalifeh, William D’Hoore, Christiane Saliba, Pascale Salameh, Marie Dauvrin

**Affiliations:** ^1^Institute of Health and Society (IRSS), UCLouvain, Brussels, Belgium; ^2^Legal Way for Advocacy and Research, Beirut, Lebanon; ^3^Faculty of Public Health—Section 2 (CERIPH), Lebanese University, Fanar, Lebanon; ^4^School of Medicine, Lebanese American University, Byblos, Lebanon; ^5^Institut National de Santé Publique d’Épidémiologie Clinique et de Toxicologie-Liban (INSPECT LB), Beirut, Lebanon; ^6^Department of Primary Care and Population Health, University of Nicosia Medical School, Nicosia, Cyprus; ^7^Faculty of Pharmacy, Lebanese University, Hadath, Lebanon; ^8^Belgian Health Care Knowledge Center, KCE, Brussels, Belgium

**Keywords:** health inequalities, healthcare bias, displaced Syrians, Lebanon, discrimination, access to health services, universal health coverage

## Abstract

**Introduction:**

According to Lebanese official data, Lebanon hosts over 1.5 million displaced Syrians (DS). Research shows that migrants encounter barriers when accessing healthcare. The social determinants of health (SDOH) related to migration are an additional challenge for DS in Lebanon, though bias plays a significant factor in exacerbating health inequalities. This study aims to identify DS perception of healthcare biases in the Lebanese healthcare system, and its consequences on DS’ accessing and receiving quality healthcare in Lebanon.

**Methods:**

A qualitative analysis using in-depth, semi-structured interviews was utilized. 28 semi-structured interviews were conducted with doctors (*n* = 12) and nurses (*n* = 16) in 2021. Six group interviews were conducted with DS (*n* = 22) in Lebanese healthcare facilities. The recruitment of participants relied on reasoned and targeted sampling. Thematic analysis was performed to identify common themes in participants’ experiences with DS accessing Lebanese healthcare.

**Results:**

The findings indicated that there were barriers to accessing healthcare related to the SDOH, such as transportation and financial resources. The results also suggested that DS perceived health biases, including discriminatory behavior from Lebanese healthcare providers, stereotypes and racism leading to health inequalities.

**Conclusion:**

Based on the perceptions and experiences reported by participants, the underlying causes of biases are due to the fragility of the Lebanese healthcare system when facing a humanitarian crisis as well as a collapsing infrastructure torn by past wars and the current socio-political and financial crises in the country. Global initiatives are required to provide the necessary resources needed for offering equitable health services. Such initiatives involve addressing biases, health inequities, discrimination, and the lack of a Lebanese infrastructure system for the provision of healthcare. Addressing health inequalities remains a major health objective in achieving health equity on the micro level (cultural awareness and competencies) and macro level (equitable distribution of resources, implementation of a universal health coverage) in order to guarantee quality healthcare services to DS.

## 1 Introduction

In recent years, global migration and forced displacement are the result of conflicts, persecution, human rights violations, violence, climate change, political oppression, and economic downturns (1). The process of adjusting to a new culture in a particular setting and in a specific environment has had an impact on the lives of the migrating and displaced populations; this includes the loss of social support networks, religious practices, and cultural norms, which causes social transformation in both physical and sociocultural spaces (2).

The process of adaptation to and integration into a new society requires social reorientation at different environmental, cultural, and professional levels as well as restructuring community and family relationships (2). In 2020, the International Organization of Migration (IOM) report highlighted the complex relationship between health and migration as an additional challenge facing health systems worldwide when achieving health equity (3). According to WHO, this is consistent with the SDOH framework on health outcomes affected by daily life conditions and systems (4). To achieve health equity, inequalities and barriers to health services must be removed. These include poverty and prejudice and their effects, such as helplessness and lack of access to good jobs with fair pay, adequate housing, education, and healthcare (5). The term “health inequalities,” used to describe variances or discrepancies among groups (6) (7), refers to structural differences among people in different socioeconomic situations in a specific territory at a certain point in its history and economic development (8). In contrast, “health inequity” or “health disparity” is a specific type of health inequality that indicates an unjust difference in healthcare that could be avoided by reasonable means (6).

Overall, studies indicate that migrants face many barriers when trying to gain access to healthcare. Such barriers result from lack of knowledge (erroneous transmitted knowledge, low health literacy, …), language barriers, different expectations and experiences of healthcare services, costs, and long delays when accessing healthcare services (9) (10) (8). Such challenges may also impact healthcare providers’ attitudes who find it difficult to deliver health services to migrants due to the poor quality of communication during health examination as well as difficulty in delivering centered and personalized healthcare services (8) (10). In turn, complex administrative procedures and discrimination defined as unjust treatment based on one’s physical appearance or cultural belonging (11) are further factors that can hinder access to healthcare (3). Research suggests that the attitudes of healthcare providers and their negative stereotyping of migrants along with lack of awareness regarding sociocultural differences have had significant implications on the provision of health services (12). Refugees, immigrants, and asylum seekers may also become victims of discrimination due to language and dialect barriers, religion, race, and their mental and physical conditions (9, 13, 14). Furthermore, negative attitudes toward people seeking care can contribute to care bias, which is defined as the ‘tendency to favor one group over another’ (15). These can include lack of respect (13), threats or physical aggression (16), or a prejudicial and unjust management of care (17). As a result, the relationship between healthcare providers and patients can be undermined due to lack of trust and a sense of isolation the patients may feel, leading to an interruption of care access (13, 18). Thus, discrimination is an essential determinant of health inequities that can impact migrants mentally and physically, leading to stress, depression, and anxiety (16, 19). It may jeopardize the provision of quality care and affect the patient’s quality of life (18).

### 1.1 Background and research questions

Lebanon ranks seventh among countries hosting refugees (3). With a ratio of 156 displaced per 1,000 inhabitants, it has over 1.5 million DS, who represent 25% of the Lebanese population (20). Lebanon is still following the 1951 Geneva Convention and has not granted refugee status to people fleeing the war in Syria. This ties in with historical and legal causes relating to the presence of Palestinians in Lebanon since1948 and the result in demographical changes and consequences leading to the Lebanese war (1975) (21–23).

Officially, the word “displaced” rather than “refugee” designates populations seeking protection (24). The most obvious example is the substitution of the Arabic word “lajii” (refugee) with “nazih” (displaced). The “nazih” is a person who leaves one region for another within the borders of a single state. The “lajii” is a person that crosses state borders and decamps from one country to another, which is the case of Syrian refugees. Lebanon reaffirms refusing any form of asylum request and reserves itself the right to review at any time the legal status of the presence of Syrian refugees on its territory. Additionally, the term “nazih” (displaced) preserves the right of displaced individuals to return to their country of origin (21).

Since October 2019, Lebanon has been witnessing severe civil and economic turmoil aggravated by the Beirut blast on August 4, 2020. The population was downgraded to a lower middle-income level by the World Bank, aggravating the country’s long-term structural weakness which already included poor public financial management, significant macroeconomic imbalances, and deteriorating social indicators. Currently half of the Lebanese population live below the poverty line due to the currency losing over 90% of its value against the dollar. The cost of living has risen dramatically, energy costs have soared, power outages have been recurrent, medical supplies have shrunk, and government services halted due to lack of funds (25–27).

For the past decade, the Lebanese Ministry of Public Health (MOPH) has made significant efforts regarding DS’ access to care with the support of the United Nations High Commissioner for Refugees (UNHCR) and other non-governmental humanitarian organizations. The MOPH has established primary healthcare centers across Lebanon to provide comprehensive healthcare services to Syrians and other vulnerable populations at affordable rates (20). Despite the concerted efforts of national and international organizations, DS still face many challenges affecting their access to primary care services (20). Providing health services is particularly challenging in Lebanon because the health system is private, fragmented, and has multiple barriers, including high costs and transportation issues (8, 28). The Lebanese economic downturn, the collapse of the health system, and the 2020 Beirut blast have triggered social tension between the Lebanese and DS communities (29). As a struggling host country with limited resources, Lebanon has limited access to quality care for its own citizens and to DS (30).

An exploratory study conducted in August 2020 aimed to examine cultural differences and discrimination as difficulties encountered by DS when using the Lebanese healthcare system and to evaluate the equity of DS access and utilization of health services in Lebanon. The results indicated that multiple challenges have jeopardized DS health and welfare. Healthcare services provided to DS perceived to be of poor quality due to inequitable access to the health system and the discriminatory behavior of healthcare providers (31). Following the identification of the problem of healthcare biases experienced by DS, the research questions were expanded as follows:

1-What types and causes of biases prevail in Lebanon when providing healthcare services to DS?

2-How does discrimination impact the access to and provision of health services to DS in Lebanon?

3-What is the impact of health inequalities on the health of DS?

## 2 Materials and methods

### 2.1 Study design and sample

Between June 2021 and August 2021, a qualitative study was conducted to gather data by interviewing healthcare professionals (HCP) and DS. The provinces included in the study were Akkar (North), Baalbek-El Hermel (North-East) and Zahle (East). These areas were chosen as they are familiar to us through our contacts and (1) because hospitals and primary care facilities there are close to DS refugee camps in Bekaa and North Lebanon. (2) The exploratory study conducted prior to this study (August 2020) (31) was also carried out in these provinces, though with different participants.

Inclusion criteria were set for the three categories of participants (refer to Table 1 Inclusion criteria of study participants): Lebanese HCP providing health services to DS in public hospitals and primary health care (PHC) centers; Lebanese HCP not providing health services to DS and working in private hospitals and clinics; and DS residing in Lebanon for at least three months. Qualified physicians and nurses were included because they are the main HCP in direct contact with DS patients.

**Table 1:** Inclusion criteria of study participants.

Healthcare providers (HCP)		Displaced syrians
providing health services to DS	not providing health services to DS	
Qualified Lebanese physicians and nurses working in primary care settings and hospitals that provide health services to DS in the selected provinces. -Enrolled participants have over two years’ experience in providing care to DS (to ensure a comprehensive understanding of the healthcare needs and challenges faced by this specific population during the economic crisis).	Qualified Lebanese physicians and nurses working in private clinics and hospitals located in the selected provinces. Enrolled participants have over two years’ professional experience.	DS aged 18 years and above living in Lebanon for at least three months (to have the opportunity to visit a healthcare facility after their displacement). Registered or not in UNHCR records but entitled to health services provided in the selected healthcare settings. Present at the time of the interview in the indicated healthcare settings located in the selected provinces.

The sample was convenient and purposeful. It was selected based on the presence of volunteer Lebanese HCP and DS in the facility at the appointment time. It involved selected participants that meet the predetermined criterion sampling (as shown in Table 1) in order to help researchers studying the provision of healthcare services in Lebanon. Included participants engaged in informed consent to be enrolled in the study (28).

In total, we conducted 28 individual interviews with HCP, including 18 working with DS and 10 not working with DS, as shown in Table 2 Characteristics of HCP participants (*N* = 28). Interviewed physicians were urologists (*n* = 2), gynecologists (*n* = 1), internal medicine practitioners (*n* = 2), general medicine specialists (*n* = 3), gastroenterologists (*n* = 1), emergency unit specialists (*n* = 2), general surgeons (*n* = 1).

**Table 2:** Characteristics of HCP participants (*N* = 28).

		Lebanese HCP working with DS (*n* = 18)		Lebanese HCP not working with DS (*n* = 10)	
		*N*	%	*N*	%
Socio-demographic characteristics					
Age	19–29	4	22.2	2	20.0
	30–49	12	66.7	8	80.0
	50 +	2	11.1	0	0.0
Sex	F	10	55.6	6	60.0
	M	8	44.4	4	40.0
Profession	Medical Doctor	8 (M1 to M8)	44.4	4 (M9 to M12)	40.0
	Registered Nurse	10 (N1 to N10)	55.6	6 (N11 to N16)	60.0
Professional Experience	2–5 y	5	27.8	2	20.0
	6–10 y	3	16.7	4	40.0
	10 + y	10	55.6	4	40.0
Professional Experience with DS	2–5 y	7	38.9	Not applicable (n/a)	
	6–10 y	4	22.2	n/a	
	10 + y	7	38.9	n/a	
Governorate/Caza	Baalbeck-Hermel	7	38.9	–	–
	Akkar	7	38.9	–	–
	Zahle	4	22.2	–	–
	Beirut	–	–	4	40.0
	Mount Lebanon	–	–	6	60.0
Workplace	Hospital	4	22.2	7	70.0
	PHC	14	77.8	-	-
	Private Clinic	–	–	3	30.0
Work Schedule	Part-time	4	22.2	7	70.0
	Full-time	14	77.8	3	30.0
Number of DS consulted by HCP per day	1 à 10	0	0.0	n/a	
	11–20	2	11.1	n/a	
	>21	16	88.9	n/a	

In parallel, 6 group interviews were carried out with a total of 22 DS participants, as shown in Table 3 Characteristics of DS participants (*N* = 22).

**Table 3:** Characteristics of DS participants (*N* = 22).

Socio-demographic characteristics		N	%
Age	18–29	11	50.0
	30–49	9	41.8
	50 +	2	9.1
Sex	F	14	63.6
	M	8	36.4
Registered with UNHCR	Yes	16	72.7
	No	6	27.3
Duration of settlement in Lebanon	3 months-1y	0	0.0
	2–5 y	1	4.5
	>5 y	21	95.5
Work in Lebanon	Yes	4	18.2
	No	18	81.8
Governorate/Caza	Baalbeck-Hermel	8	36.4
	Akkar	7	31.8
	Zahle	7	31.8
Place of residence	Camps	16	72.7
	Appartement	6	27.3
Number of visit(s) to healthcare facilities	1^st^ visit	0	0.0
	2–5 visits	1	4.5
	>5 visits	21	95.5
Receiving contributions from UNHCR and NGOs	Yes	16	72.7
	No	6	27.3
Types of contributions received	Food	8	36.3
	Financial	7	31.81
	Both	7	31.81

### 2.2 Ethical aspects

The Institutional Board Review (IRB) of Al Rahma Hospital approved the study under reference number 077–2021. The study complies with the Declaration of Helsinki. Participants were aware they could withdraw from the study any moment and that their participation was voluntary, anonymous, and without incentive. Before starting the interview, participants were informed that this research was part of a doctoral study. The objectives of the study were made clear to them. They were also told that the interview would be recorded but would remain confidential and used solely for the purpose of the research before being deleted.

### 2.3 Data collection

To gather data, group interviews with DS and semi-structured individual interviews with HCP (whether or not they worked with DS) were conducted. The aim was to identify DS perception of healthcare biases in the Lebanese healthcare system and describe the consequences of such biases on DS’ accessing and receiving quality healthcare during the Lebanese economic crisis.

Kohn and Christiaens (2014) (32) consider that semi-structured individual interviews are justified when case-sensitive and highly personal questions are part of the study (32). This type of interview allows participants to share their experiences, perception, feelings, and impressions about specific events (33, 34). The interviews were confidential and anonymously conducted to give participants freedom of expression when voicing their concerns.

The group interview was selected [1] because DS tend to seek medical care as a family and [2] family members accompany women to medical visits. This feature allowed us to enroll homogeneous groups (family members) as well as heterogeneous groups (different families and friends with similar experiences). Moser and Korstjens (2017) (34) estimate that small groups of six participants allow more time to express their points of view and give detailed information. The exploratory study conducted in August 2020 (31) revealed that group interviews provide relevant insights based on shared experiences.

The interviews with DS were conducted in Arabic using lay language to ensure clarity and eliminate confusion and misunderstanding. Those carried out with HCP were formulated in English or French using medical verbatim. The average duration of an interview was 50 min with the HCP and 60 min with the DS. All participants gave their oral consent prior to the interview.

### 2.4 Interview guides

Three semi-structured interview guides were prepared for each category of participants. They were formulated in French based on the objectives presented in Table 4 Objectives of the interview guides, then translated into Arabic using backward and forward translation. The interview guides were tested and validated in June 2021. We visited the PHC centers in Akkar and interviewed HCP working with DS (4 interviews) and a group of DS seeking care in the facility (2 interviews). We also interviewed HCP working in a hospital in Beirut that was not offering health services to DS. A report detailing the results of the pilot interviews was established, shared, and discussed with other authors before proceeding. Participants understood the questions and felt comfortable during the interview. They showed interest in the theme of the study and provided positive feedback. Participants were informed at the start that the interview would last approximately one hour. As the pilot study did not introduce changes to the interview guide, the data collected from the interview guides tests were added to the confirmatory sample, except for one with a HCP which lasted only 15 min during which the participant answered only part of the questions.

**Table 4:** Objectives of the interview guides.

Interview guides			
N^o^	Target Population	Type of the Interview	Objectives
**1**	**HCP working with DS**	Semi-structured individual interview	The interview guides consist of three parts: A first descriptive and normative part including socio-demographic characteristics of the target population with a general overview of healthcare biases. A second prospective part encouraging participants to share their opinions on existing situations in order to identify results and solutions and resolve the issue of biases in Lebanese healthcare. A third part addressing the effect of the Lebanese context and its impact on the work of health professionals and the lives of DS.
**2**	**DS**	Group interviews	
**3**	**HCP not working with DS**	Semi-structured individual interview	The interview guide includes a first part for socio-demographic characteristics, another for questions on the access to care and the interaction of health professionals with patients during the delivery of care, and a third part aiming to understand whether the Lebanese context has an influence on patients’ diagnosis or treatment.

### 2.5 Analysis and trustworthiness

Each interview was anonymized before transcription for people and places. The participant received a unique identifier. For HCP, “M” designates a “medical doctor,” “N” refers to “nurse,” and “DS” a “Displaced Syrian.” The last letter indicates the number of participants. HCP working with DS are labeled as follows: M1 to M8 and N1 to N10. As for the HCP not working with DS, the acronyms were: M9 to M12 and N11 to N16.

A first and second floating listening sessions were performed to obtain a transcript fully compatible with the recordings. The translation into both French and English was carried out by professional trilingual translators and then confirmed by the research team. This triangulation guaranteed the credibility of the translation and the trustworthiness as to the cultural sensitivity of the questions.

A qualitative content analysis of the transcripts was carried out. This approach is well-known in health care science and is often used for interpreting text material (35).

The structured approach of content analysis allows a descriptive view of the HCP and DS situation from multiple data sources and perspectives. Thematic analysis was used to identify common themes in participating DS experiences when accessing Lebanese healthcare. The analysis of the data used an inductive-deductive approach to generate thematic categories. Based on the interview guide, a provisional category system was created. It consisted of the current healthcare situation and healthcare needs (deductive approach). In the course of the analysis, this was adapted according to the content of the transcripts and supplemented by emerging new categories (inductive approach). Each transcript was coded into main categories and subcategories by three independent researchers (RK, MD and CS) and discussed in consensus meetings with a fourth researcher (WD). Both across-case analyses and within-case analyses were done to gather common categories for HCP and DS. Quotes were used to illustrate each category and the same analysis was used for individual and group interviews. The description of themes is mentioned in Table 5 Description of themes and detailed in the results section.

**Table 5:** Description of themes.

Themes	Description	Research Questions Targeted
Provision of health services in Lebanon	Several administrative, logistics or financial barriers exist when accessing care.	1,2,3
**Socio-cultural context and practices**	Socio-cultural differences between the Lebanese and Syrian populations influence the interactions during the delivery of care and create tension between caregivers and patients, which affects the perceived quality of the care delivered.	2
**Bias in health services offered to DS**	Discriminatory behaviors were detected when providing care to DS. Both participants, HCP, and DS, reported from their own perspectives the underlying causes and their effects on the health of DS.	1,2,3
Effects of the Lebanese crisis (since October 2019) on the provision of health services	Affected by the crisis, participants shared their feelings and emotions. They discussed the adverse effects of the crisis on health care in Lebanon and the worsening biases in care due to the crisis.	1,2,3

Several measures were taken to ensure the trustworthiness of the study (36). The research team ensured the research questions were clear and precise. The interview guides were validated by conducting interview tests with three types of participants prior to the study. The research team reviewed the content of these interview tests, advised on minor changes, and gave approval to proceed with the interview guides. Data validity was guaranteed via person triangulation, data collected from three different sorts of people and their varied perspectives. DS and healthcare professionals, working or not with DS, were included in the study to ensure that a range of perspectives was represented. The interviews were conducted in a neutral and non-judgmental manner, with open-ended questions that encouraged participants to share their experiences and opinions. A peer debriefing on qualitative basis was used with knowledgeable peers and researchers. Thematic analysis and coding were used together with other well-established research techniques to properly evaluate and interpret the interview data. The research team then examined and confirmed the results to make sure they were reliable and that they accurately reflected the experiences and viewpoints of the participants. Redundancy and data saturation were achieved in the absence of any new information.

## 3 Results

### 3.1 Provision of health services in Lebanon

HCP mentioned that DS visit primary care facilities for healthcare provision because they are free of charge and offer multiple health services, such as vaccination, medical consultation, imaging, laboratory testing, and medications.

“DS come to our PHC in Akkar because they benefit from free health services for only 3000 Lebanese pounds, an insignificant amount. They get vaccinated and receive medication for free, such as Panadol for children suffering from post-immunization fever. Hence, they get the most out of the services that are offered.” (N1)

Nevertheless, these services are not always free of charge. DS can find difficulty paying medical consultation fees and specific medical services. Participants stated that the economic downturn in Lebanon aggravated the situation.

“Consultation fees are increasing; we can’t even afford to pay the 3000 Lebanese pounds.” (DS5)

In some cases, DS returned to Syria to benefit from health services offered free of charge. Such a move could compromise their health and lead to hazardous outcomes.

*“My neighbor returned to Syria for an open-heart surgery because he can’t afford the expensive hospital charges in Lebanon.”(DS2)*

According to the interviewees (DS and HCP), financial challenges arise when patients transfer from PHC to hospital for a one-day stay or ambulatory care. In such cases, DS cannot afford the charges and have to confirm in writing their own consent not to receive health services even when their condition is critical. Refusing to receive care can also impact HCP as care givers and jeopardize their profession and medical responsibility toward the patient.

HCP mentioned that certain hospitals, abiding by internal regulations, prioritize Lebanese citizens over DS when benefiting from health services.

“DS are finding difficulty being admitted to hospital because the priority is for Lebanese citizens rather than Syrians.” (M1)

HCP not working with DS spoke about the challenges Lebanese patients face when trying to be admitted to hospitals. They mentioned how the financial and economic crisis was compelling them to visit hospitals only for very urgent situations. Otherwise, they skip all consultations, routine checkups, and blood tests.

“Most patients no longer visit for consultations, routine check-ups, or blood tests. In view of the financial crisis, they cannot afford to pay the significant fees and costs. We only witness very urgent and critical cases of patients coming to hospital …” (M10)

### 3.2 Socio-cultural context and practices

HCP shared their experience of DS girls getting married and pregnant at an early age and considered this as an unusual occurrence in the Lebanese community.

“We are faced with cultural particularities of the DS community, mainly marriage at a very young age, polygamy, large families, and submissive women. We also encounter divorce at a young age. Uses of contraceptives and sharing emotions and feelings are taboo. All of this is an odd culture to the Lebanese society.” (M7)

Female DS prefer being consulted by a female physician. This choice is made out of a socio-cultural practice.

« *I visit a female doctor … Like all other Syrian women …I cannot get undressed during a medical consultation …* ». (DS12).

Results of the interview showed that HCP considered DS careless regarding their children’s health. Despite all their effort to educate them and increase awareness of the importance of vaccination and medical follow-up, HCP estimate that DS are not cooperative. Such situations create tension during the provision of care.

In other respects, cultural and health beliefs are the leading cause of conflicts and tension between HCP and DS. HCP sometimes perform unnecessary procedures or administer a treatment to avoid DS complaints to UNHCR and Non-Governmental Organizations (NGO). Physicians consider such situation compromising on the professional, medical, and ethical levels.

“DS have a collective viewpoint about treatment. They consider antibiotics and getting an injection a mainstay. They estimate it is appropriate to get these treatments and judge the physician as incompetent if he doesn’t prescribe them. […]. Sometimes, HCP are compelled to prescribe an antibiotic or an injection to avoid DS complaining to NGOs.” (M4)

HCP say DS are try to benefit from free-of-charge prescribed medication in Lebanese PHC. In Syria, health services are free of charge. These differences between both health systems lead to tension between patients and HCP, which can affect the quality of health services.

“Sometimes DS start yelling for an antibiotic even if they don’t need one … In other terms, they abuse the system and claim their right to get the antibiotic just because it is free!”(N5)

### 3.3 Bias in health services offered to DS

#### 3.3.1 Forms of healthcare bias

DS reported experiencing different forms of biases in the Lebanese system. Discrimination and prejudice are revealed through difficulty in accessing health services, refusal of hospital admission, and extended waiting time. Claims such as “*You cannot be admitted to hospital because we do not have additional places for Syrians*” are interpreted as a form of hatred toward Syrians, which is also a form of health inequity.

HCP estimated that this negative feeling toward DS is due to the scarcity or lack of health system resources. As a result, Lebanese citizens are forced to pay for health services.

“They want to live in our country and benefit from our health services for free … and Lebanese have to pay to get the same services! I am clear, we do not want them. DS want to live in our country, share our resources, and get access to health services that are entitled to us and on top of that they do not pay any fees.” (M4)

DS claimed they are not getting the same quality health services as Lebanese. This discrimination was revealed in the DS’ perception through the inappropriate ways HCP communicate with them.

“HCP act differently with a Lebanese patient. They communicate with us in an inappropriate and impolite manner. We feel that they don’t make enough effort to help us.” (DS4)

#### 3.3.2 Causes of healthcare bias

In their comments, HCP confirmed that political causes could be behind the tension felt with DS. It all goes back to a history of conflicts during the hegemony of the Syrian army over Lebanon (1943–2005). Another reason for tension was the need for the Lebanese to share limited and scarce resources with Syrians. For interviewed HCP, DS are perceived as a burden in a struggling country where Lebanese citizens are not benefiting from their rightful use of their country resources while DS are.

“We are living in a country in crisis, where there is no medication, no food, no gas … and what worsens the situation is the presence of DS. Tell me how you can accept a DS receiving medication for free when the Lebanese are in need and cannot afford to pay for medication?”(N6)

HCP also considered that DS themselves are triggering such conflictual behavior because of their arrogance, disrespect, and cynical attitude.

“Bias exists because DS are impulsive and stubborn. They are the ones who trigger HCP's negative behavior.” (N4)

HCP estimated that another cause of discrimination is tied to the high number of DS visiting PHC to benefit from the health services provided. They sometimes felt overwhelmed and stressed. This statement was only expressed by PHC who usually receive over 20 Syrian patients a day.

From their side, DS said that “*Lebanese people in general and HCP in particular do not like Syrians because of the politically tense history between the two countries.”* Therefore, they felt discriminated against and rejected.

Moreover, the interviews showed that the current situation in Lebanon is the leading cause of discrimination, rejection, stigmatization, and bias in health services provided to DS. HCP considered that the political and economic downturn in Lebanon is exacerbating health inequality. DS are getting financial aid that improves their living conditions while Lebanese citizens are struggling during the severe crisis. As a result, Lebanese felt ‘displaced’ and left without support in their own country, while DS were getting multiple benefits and support from international organizations.

*“Honestly, the Lebanese are the ones who are “displaced” in their own country and Syrians are more at ease and in a better situation … Ironic, isn’t it? All countries support DS! But what about the Lebanese? I see them trying to find food in garbage cans. It is a shame!” (N6)*

#### 3.3.3 Impact of bias on the provision of health services

The interviews also showed the detrimental effect of biased healthcare on diseases. HCP stated that DS who refused medical consultation to avoid stigmatization were putting their health at risk and encouraging the dissemination of diseases mainly during epidemics, more particularly during COVID-19.

Certain HCP revealed that they could not stand the patients’ unpleasant odor and were forced to carry out a quick medical examination, which could result in a potentially erroneous diagnosis.

“I try to finish fast with their medical consultation and avoid doing a complete check-up because I cannot stand the bad smell of certain DS … In certain situations, DS refuse to get undressed or remove their shoes to avoid my reaction … As a result, the diagnosis may be erroneous due to an incomplete physical examination.” (M6)

HCP stated that DS self-medicated themselves to avoid biased medical treatment. They would sometimes get inappropriate drugs to treat their condition, knowing that a lot of medication is purchased over the counter or without a prescription in Lebanon.

Health inequality can also lead to hazardous outcomes mainly in post-surgery. Follow-up of a Lebanese patient is different after a surgical procedure.

“I have witnessed cases where DS were discharged from hospital following a surgery even if they have complications that may put their life at risk … It is not the case with Lebanese patients!” (M7)

“They asked me to leave the hospital directly after surgery. When my wife wanted some clarification, she understood the bed was reserved for a Lebanese patient …” (DS15)

HCP not working with DS stated that the care provided complies with professional ethics regardless of the patient’s socio-economic status.

“There is no difference in our relationship with patients … We offer our care in the same way to all patients … Personally, my work is not influenced by the status, socio-economic level, or religion of the patient.”(N14)

### 3.4 Effects of the Lebanese crisis (since October 2019) on the provision of health services

Whether or not they worked with DS, the HCP interviewed revealed they were affected by the situation in Lebanon. The economic, political, and financial crisis is described as “severely hitting” healthcare institutions. In the interviews, HCP expressed many negative emotions, such as fear, anxiety, frustration, depression, demotivation, anger or even sadness, which made them think about leaving the country to look for new opportunities abroad.

“I am anxious and stressed because of this situation … Our salaries are worth nothing … I don't know how we can keep working.” (N1)

From their side, the DS interviewed said they were living in very difficult conditions and were negatively affected by the crisis despite receiving food and financial contributions.

“The situation is very difficult at the moment…the 400,000 Lebanese pounds (financial contribution received from UNHCR) is worth nothing… We can hardly feed ourselves.” (DS3)

Whether or not they worked with DS, the HCP interviewed also pointed out the negative impact of the economic, financial, and political crisis on caregiver-patient relationship. They stated that the situation was critical and patients were seriously affected by the crisis due to shortages of drugs, fuel, and hospital equipment, which was affecting the entire health sector and making the delivery of care difficult.

“It's true that the situation is miserable… But we have no choice but to help people and do our best to provide them with satisfactory services… We don't know the situation of each patient, but we understand well that there are many poor people who need help … Shortage of medication and lack of equipment make the situation even more complex.” (M8)

Though confident and aware of their responsibility to carry out their tasks in a professional manner regardless of arising situations and events, interviewed HCP feel downhearted. According to their statements, the impact of the current situation on the provision of care was undermining their relationship with patients and complicating their task, particularly when patients become increasingly demanding and unbearable.

“Being healthcare professionals, we have a mission to help people regardless of their situation… but it's still hard… Patients have become unbearable. I understand them, but I too suffer from this situation.” (M12)

They also reported that medical and nursing staff are leaving hospitals and that a drain of skills can only weaken and harm the health system and the delivery of care.

“I don't know if we will always find doctors in hospitals due to this 'brain drain'… Competent doctors are leaving the country and so are nurses… The consequences are dramatic on the health system.” (M11)

### 3.5 Factors leading to healthcare bias and inequalities in the provision of health services to DS in Lebanon

**Figure 1:**
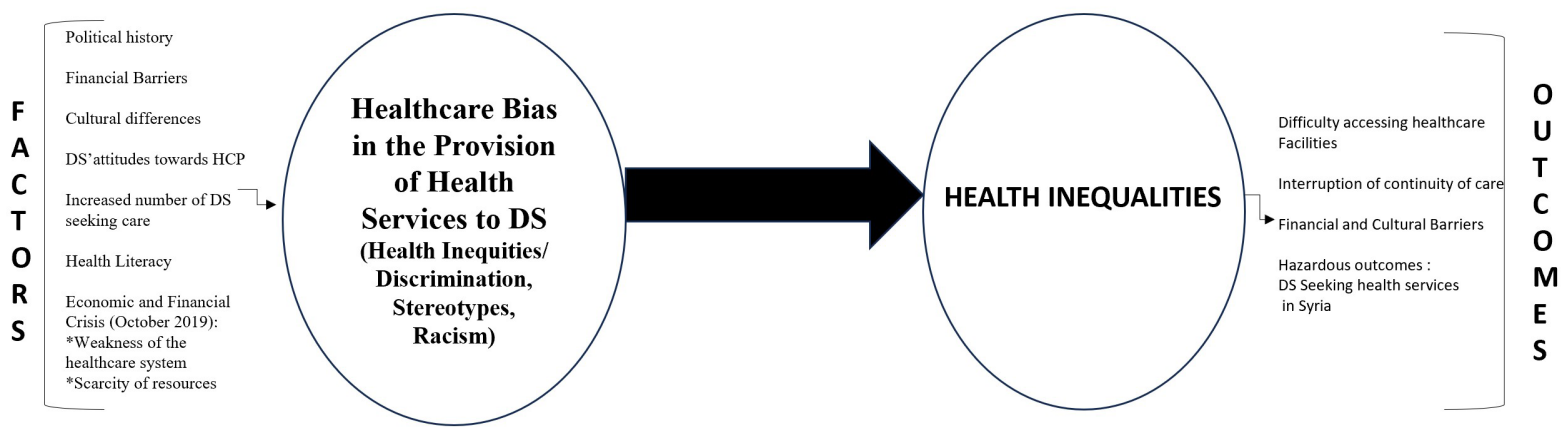
Factors leading to healthcare bias and inequalities in the provision of health services to DS in Lebanon.

Based on the findings, several factors contributed to health care bias in the Lebanese system. As shown in Figure 1, health inequalities are considered as the result of healthcare bias and inequities and have an impact on the quality of care provided to DS in Lebanon.

## 4 Discussion

The results showed that DS are facing numerous challenges in accessing and using healthcare services in Lebanon. The results addressed the research questions by identifying the factors that lead to bias, based on the perceptions and experiences reported by HCP and DS.

### 4.1 DS’health outcomes

The SDOH framework notifies that health outcomes are determined by conditions and systems in daily life (4). Hence, the daily life conditions of migrating DS contribute significantly to differences in health outcomes and unequal access to care. In general, migrants are less wealthy and have worse health conditions than the native population (37). The SDOH, including the current economic crisis, high fuel costs, limited transportation means, political instability, and low security in some regions have contributed to an increase in health inequalities. Faced with these complexities, DS tend to delay or avoid seeking care, or even choose to return to their native country where health services are offered for free. Such decisions can be detrimental to their health and well-being (38). Highlighting barriers to health care and SDOH for DS communities is particularly important because the economic downturn in Lebanon has underscored health disparities among this population.

Additionally, DS mentioned that HCP are constantly in a rush when delivering care and prioritize Lebanese patients. This aligns with earlier research that found that minority and displaced patients felt rushed or ignored by healthcare professionals during clinical encounters (39–42). In other studies, some patients complained they were unfairly skipped over by patients perceived as privileged (43–45). Thus, the perception of prioritizing Lebanese citizens over DS to benefit from health services plays a significant role in exacerbating these challenges. Yet the results of the study, which are consistent with other results (25, 27), reveal that DS’s experiences of access to healthcare services are better and simpler than Lebanese who must go through lengthy permission processes to receive healthcare services and who must also pay expensive fees. HCP also estimated that the situation of DS is far better than that of Lebanese citizens due to the continuous financial and logistic support provided by NGOs and international organizations. Thus, DS can receive health services at lower costs than those paid by host citizens.

### 4.2 Socio-cultural differences as barriers to healthcare services

The findings of the study also pointed out to socio-cultural differences between the two populations. The cross-cultural relationship between HCP and DS is similar to that of immigrants in other contexts in the literature. The disappointed attitude of DS toward Lebanese HCP aligns with that revealed in a study conducted in Norway showing that immigrants from Sub-Saharan Africa are disappointed and frustrated with their relationship with Norwegian doctors and prefer to be consulted by a fellow citizen. They are convinced that a doctor from their own ethnic background would understand, respect, and treat them better than Norwegian doctors (12).

While cultural differences between Lebanese and Syrians may be less prominent in some provinces of Lebanon, they still create tension between HCP and DS, which leads to challenges during the provision of care. An example of this is the governorate of Akkar, located in the north of Lebanon, where DS share common socioeconomic situation and traditions with the hosting community (46).

According to the findings, HCP also consider that the DS perception of health, vaccination, marriage, contraception, and gender differences constitute additional barriers that lead to tension between the two populations. While HCP may see early marriage as an odd occurrence in the Lebanese community, Syrians consider it as a coping strategy and a way to protect young girls and secure their future (47, 48).

In other respects, cultural differences in health beliefs between HCP and DS may create additional obstacles and challenges during the delivery of care. Although Syrians seeking healthcare from HCP expect to receive adequate treatment, some still use religious or supernatural healing methods in parallel. Seeking health services can be hindered by the idea that God is ultimately the only healer (47). In Syria, socioeconomic, ethnic, and religious diversity within the population has significant influence on the dynamics of the community, health decision, beliefs, and attitudes (47).

### 4.3 DS ‘perception of healthcare bias and discrimination

On the other hand, the findings of the study, which also aligns with the results of the exploratory study (August 2020) (31), show that healthcare bias is manifested in aggressive and racist communication and the adoption of segregated attitudes toward DS patients. In this context, HCP may act either consciously or unconsciously in an oppressive manner during their interactions with DS, as indicated in the literature. This results in incidents, such as racial slurs, lack of empathy or respect (49, 50), provision of inferior care, refusal to treat a patient, marginalization, and long waiting times (9, 51). Yet our results revealed some exceptions with one doctor in Baalbeck-Hermel showing compassion toward the DS and encouraging other doctors to assist this vulnerable population. Prior to the Syrian war, the aforementioned doctor used to travel to Damascus and provide medical care to Syrians. He forged close ties with them and viewed them as victims of the conflict there.

The causes of healthcare bias toward DS in Lebanon largely described in this study complement the results of the exploratory study conducted in August 2020 (31). The particularities of the Lebanese context relate to a history of political conflicts between the two countries as well as the current economic and political crisis in Lebanon that has made resources rare and strained the health system. Recent study conducted by Kikano et al. (2021) (52) shows that Lebanese felt dispossessed and in competition with DS because Syrians have been replacing Lebanese workers in low-skilled jobs. This situation increases the resentment Lebanese people feel toward them (52). These reasons may contribute to HCP’s negative attitudes, which have a potential impact on DS physical and mental health. Experiences of discrimination may also trigger improper health behaviors, such as avoiding seeking health care. Loss of trust in the quality of health services provided and patient dissatisfaction can also lead to a detrimental physical and mental impact among those in need of healthcare (53, 54).

The findings of the study, in line with other studies (25, 52), confirm that Lebanon’s resource shortage is a major factor in the discrimination that DS experience when seeking healthcare services. Lebanese citizens and DS receive unequal distribution of resources and services due to rationing and priority. This is specifically a result of the healthcare system strained capacity, which makes it difficult to meet the needs of all healthcare seekers. However, because the Lebanese healthcare system is largely privatized, DS find it challenging to receive necessary medical care owing to their financial limitations, which results in detrimental effects on their health. Previous studies showed that language, socio-cultural differences, and discrimination also led to avoidance of emergency healthcare, which results in harmful consequences on refugees’ health (10, 18, 55).

Similarly, DS confirmed that the main issue of accessing health services is discrimination. They considered they were the victims of hostility, lack of respect, and prejudice. They claimed that they felt ignored and neglected and that their treatment was inappropriate. They also denounced carelessness in the way HCP interacted with them. This appears to be connected with HCP’s inadequate communication with the DS due to their discriminating and stereotypical viewpoints. Studies show that healthcare professionals frequently doubted the ability of refugees and other minority groups to understand information (44, 56) and did not provide them with enough information about their treatment, leaving them “in the dark” (57, 58). Refugees and migrants claimed their intelligence was diminished (44) when healthcare practitioners addressed them in an unduly straightforward manner or imposed their own opinions on them (58). Raised voices, abrupt tones, and dismissive body language used by healthcare professionals when treating refugees and migrants contributed to this view (41, 43, 59).

In the study, some geographical differences are also pointed out by DS. Comparing themselves to DS in other governorates, DS in Zahle felt more excluded and marginalized, which made them seek healthcare services in other governorates. They expressed concerns over being denied access to health facilities in this specific location due to the discriminatory practices of some HCP who refused to receive Syrians in their health facilities. They pointed out the arrogance and cynicism HCP treated them with and considered it a discriminatory activity. This aligns with the literature revealing that healthcare professionals are less sympathetic toward refugees or migrants and are often prone to losing their temper when interacting with them (60).

From their perspective, nurses and doctors working full or part time say that discriminatory attitudes are due to their being overwhelmed by the large number of DS they receive every day in their health facilities. This was only expressed by those who usually receive over 20 DS patients a day. This is made worse by time constraints healthcare professionals experience (60, 61), which force them unintentionally to limit their interactions with minority and refugee patients (62).

Moreover, Lebanese HCP said they limited their contact with DS who smelled unpleasant. Healthcare services were also affected by healthcare professionals refusing to interact with or deliver care to refugees or minority groups because of their appearance (60, 63). They admitted not providing tailored treatment to refugees as they did not understand their differing needs (41, 63, 64). Such underlying causes could eventually lead to increased tension and conflicts between minority and majority populations, which could be perpetuated by biased healthcare encounters (65).

Finally, due to their demographic evolution on Lebanese territory and the Lebanese geopolitical context, DS nurtured a feeling of being threatened, which also contributed to “hatred” toward HCP. Social psychology considers discrimination as an attitude adapted by individuals to eliminate the source of threat to the survival of the group (66). Based on the interviews results, the perception of discrimination is generalized among DS (66). It is important to emphasize that the DS perception of discrimination is not linked to HCP lacking skills but rather to a particular situation explained by the occurrence of several personal, contextual, and geographical factors leading to health inequalities toward DS in Lebanon during the provision of care.

### 4.4 Limitations of the study

A limitation should take into account the fact that 30% of the DS interviewed are not covered by the UNHCR. This figure does not seem to have a negative impact on accessing or delivering healthcare. Even when a part of the DS is not provided health coverage by the UNHCR, the interviews did not consider it an obstacle to accessing healthcare services because the interviews happened during the delivery of care.

The findings of the study have limited generalizability. The authors have attempted to improve transferability by elaborating on the context and research methods and by linking the results to existing evidence so that readers can better determine the relevance of these findings in other contexts. The study was carried out in the specific context of the northern Bekaa region, one that is densely populated by DS. It should be considered as a basis for further studies covering the whole country and verifying possible generalization.

## 5 Conclusion

Addressing health inequalities remains a major health objective in achieving health equity. It involves addressing biases, health inequities, discrimination, and lack of a Lebanese infrastructure system for the provision of healthcare.

It is a human right for all people to benefit from high-quality personalized and person-centered care, regardless of their origin, ethnicity, and social or economic status. In the Lebanese context, bias toward DS in the provision of care was exacerbated by the country’s severe economic downturn, the Beirut blast, and the ongoing political crisis (25, 52). With scarce resources, the Lebanese health system is struggling, while a growing number of DS are offered full support by international organizations. This situation constitute a huge challenge for the Lebanese healthcare system and for that of other host countries that lack time to plan, manage, and comprehend the health needs of refugees and migrants (9). NGOs and the international community are aware that national health policies that are more integrated, compassionate, culturally sensitive, and responsive to the needs of DS are urgently needed to prevent bias and improve health equity in Lebanon.

Better access to primary healthcare services is a suggestion made by MOPH and NGOs for dealing with DS medical issues in Lebanon. By increasing the number of PHC in refugee communities, MOPH seeks to ensure that all patients can pay and receive health services. The establishment of a unified health information system that gathers, analyzes, and shares data regarding DS health needs will enable evidence-based decision-making, which is crucial to effectively coordinate healthcare efforts among governmental organizations, non-governmental organizations, and international agencies.

On the other hand, in order to counter healthcare disparities and inequities that DS may face during the delivery of care, training in cultural competence should be provided to healthcare professionals to help them deliver patient-centered care and culturally sensitive.

Reducing financial risk is also vital to alleviate the burden of health spending on disadvantaged people. It is an essential step toward universal health coverage (UHC) (67). Implementing UHC helps advance equity as it guarantees access to a wide range of healthcare services, including preventive, curative, and rehabilitative care. It eliminates financial barriers by providing healthcare services to all individuals, including DS, without imposing excessive out-of-pocket expenses. UHC also minimizes healthcare gaps between various population groups and ensures that DS and Lebanese citizens have the same standard of care.

Thus, the implementation of UHC requires an expansion of healthcare infrastructure, including the establishment of additional healthcare facilities and the recruitment of healthcare professionals to meet the increased demand for services. Its implementation in a country suffering from a severe financial and economic crisis, as is the case in Lebanon, can act as a safety net relieving people of the burden of paying for healthcare and can guarantee easy access to healthcare services for both DS and Lebanese nationals. To help Lebanon’s healthcare system cope with the burden of implementing UHC, donor nations and international organizations must work together to provide financial support, enhance health system resilience, and encourage collaboration and partnership.

## Data availability statement

The original contributions presented in the study are included in the article/Supplementary material, further inquiries can be directed to the corresponding author/s.

## Ethics statement

The studies involving humans were approved by the institutional review board of Al Rahma Hospital— Lebanon (Ref: 077-2021). The studies were conducted in accordance with the local legislation and institutional requirements. Written informed consent for participation was not required from the participants or the participants' legal guardians/next of kin because oral consent was obtained prior to the interview.

## Author contributions

RK: Conceptualization, Formal analysis, Investigation, Methodology, Resources, Writing – original draft, Writing – review & editing. WD’H: Conceptualization, Formal analysis, Methodology, Supervision, Validation, Writing – review & editing. CS: Methodology, Supervision, Writing – review & editing. PS: Writing – review & editing. MD: Conceptualization, Formal analysis, Methodology, Supervision, Validation, Writing – review & editing.
