# Supplementary material for: Healthcare bias and health inequalities towards displaced Syrians in Lebanon: a qualitative study
Source: Front Public Health. 2023 Nov 30;11:1273916. doi: 10.3389/fpubh.2023.1273916 (PMC10720425; doi:10.3389/fpubh.2023.1273916)
Supplement: Supplementary file 1 [file Table_1.DOCX]

Supplementary Material

**Tables**

**Table 1. Inclusion criteria of study participants**

| **Healthcare providers (HCP)** | | **Displaced Syrians** |
| --- | --- | --- |
| **providing health services to DS** | **not providing health services to DS** |  |
| - Qualified Lebanese physicians and nurses working in primary care settings and hospitals that provide health services to DS in the selected provinces.   -Enrolled participants have over two years’ experience in providing care to DS (to ensure a comprehensive understanding of the healthcare needs and challenges faced by this specific population during the economic crisis). | -Qualified Lebanese physicians and nurses working in private clinics and hospitals located in the selected provinces.  -Enrolled participants have over two years’ professional experience. | -DS aged 18 years and above living in Lebanon for at least three months (to have the opportunity to visit a healthcare facility after their displacement).  -Registered or not in UNHCR records but entitled to benefit from health services provided in the selected healthcare settings.  -Present at the time of the interview in the indicated healthcare settings located in the selected provinces. |

Table 2. Characteristics of HCP participants (N=28)

|  | | **HCP working with DS**  **(n=18)** | | **HCP not working with DS (n=10)** | |
| --- | --- | --- | --- | --- | --- |
|  |  | **N** | **%** | **N** | **%** |
| **Socio-demographic characteristics** |  | | | | |
| Age | 19-29 | 4 | 22.2 | 2 | 20.0 |
|  | 30-49 | 12 | 66.7 | 8 | 80.0 |
|  | 50 + | 2 | 11.1 | 0 | 0.0 |
| Sex | F | 10 | 55.6 | 6 | 60.0 |
|  | M | 8 | 44.4 | 4 | 40.0 |
| Profession | Medical Doctor | 8 (M1 to M8) | 44.4 | 4 (M9 to M12) | 40.0 |
|  | Registered Nurse | 10 (N1 to N10) | 55.6 | 6 (N11 to N16) | 60.0 |
| Professional Experience | 2-5 y | 5 | 27.8 | 2 | 20.0 |
|  | 6-10 y | 3 | 16.7 | 4 | 40.0 |
|  | 10 + y | 10 | 55.6 | 4 | 40.0 |
| Professional Experience with DS | 2-5 y | 7 | 38.9 | Not applicable (n/a) | |
|  | 6-10 y | 4 | 22.2 | n/a | |
|  | 10 + y | 7 | 38.9 | n/a | |
| Governorate/Caza | Baalbeck-Hermel | 7 | 38.9 | - | - |
|  | Akkar | 7 | 38.9 | - | - |
|  | Zahle | 4 | 22.2 | - | - |
|  | Beirut | - | - | 4 | 40.0 |
|  | Mount Lebanon | - | - | 6 | 60.0 |
| Workplace | Hospital | 4 | 22.2 | 7 | 70.0 |
|  | PHC | 14 | 77.8 | - | - |
|  | Private Clinic | - | - | 3 | 30.0 |
| Work Schedule | Part-time | 4 | 22.2 | 7 | 70.0 |
|  | Full-time | 14 | 77.8 | 3 | 30.0 |
| Number of DS consulted by HCP per day | 1 à 10 | 0 | 0.0 | n/a | |
|  | 11 - 20 | 2 | 11.1 | n/a | |
|  | >21 | 16 | 88.9 | n/a | |

**Table 3. Characteristics of DS participants (*N*=22)**

| **Socio-demographic characteristics** | | **N** | **%** |
| --- | --- | --- | --- |
| Age | 18-29 | 11 | 50.0 |
|  | 30-49 | 9 | 41.8 |
|  | 50 + | 2 | 9.1 |
| Sex | F | 14 | 63.6 |
|  | M | 8 | 36.4 |
| Registered with UNHCR | Yes | 16 | 72.7 |
|  | No | 6 | 27.3 |
| Duration of settlement in Lebanon | 3 months-1 y | 0 | 0.0 |
|  | 2-5y | 1 | 4.5 |
|  | >5 y | 21 | 95.5 |
| Work in Lebanon | Yes | 4 | 18.2 |
|  | No | 18 | 81.8 |
| Governorate/Caza | Baalbeck-Hermel | 8 | 36.4 |
|  | Akkar | 7 | 31.8 |
|  | Zahle | 7 | 31.8 |
| Place of residence | Camps | 16 | 72.7 |
|  | Appartement | 6 | 27.3 |
| Number of visit(s) of healthcare facilities | 1^st^ visit | 0 | 0.0 |
|  | 2-5 visits | 1 | 4.5 |
|  | >5 visits | 21 | 95.5 |
| Receiving contributions from UNHCR and NGOs | Yes | 16 | 72.7 |
|  | No | 6 | 27.3 |
| Types of contributions received | Food | 8 | 36.3 |
|  | Financial | 7 | 31.81 |
|  | Both | 7 | 31.81 |

Table 4. Objectives of the interview guides

| **Interview guides** | | | |
| --- | --- | --- | --- |
| **N^o^** | **Target Population** | **Type of the Interview** | **Objectives** |
| **1** | **HCP working with DS** | Semi-structured individual interview | The interview guides consist of three parts:  -A first descriptive and normative part including socio-demographic characteristics of the target population with a general overview of healthcare biases.  -A second prospective part encouraging participants to share their opinions on existing situations in order to identify results and solutions and resolve the issue of biases in Lebanese health care.  -A third part addressing the effect of the Lebanese context and its impact on the work of health professionals and the lives of DS. |
| **2** | **DS** | Group interviews |  |
| **3** | **HCP not working with DS** | Semi-structured individual interview | The interview guide includes a first part reserved for socio-demographic characteristics, another containing questions on the access to care and the interaction of health professionals with patients during the delivery of care, and a third part aiming to understand whether the Lebanese context has an influence on patients’ diagnosis or treatment. |

**Table 5. Description of themes**

| **Themes** | **Description** |
| --- | --- |
| **Provision of health services in Lebanon** | Several administrative, logistics or financial barriers exist when accessing care. |
| **Socio-cultural context and practices** | Socio-cultural differences between the Lebanese and Syrian populations influence the interactions during the delivery of care and create tension between caregivers and patients, which affects the perceived quality of the care delivered. |
| **Bias in health services offered to DS** | Discriminatory behaviors were detected when providing care to DS. Both participants, HCP and DS, reported from their own perspectives the underlying causes and their effects on the health of DS. |
| Effects of the Lebanese crisis (since October 2019) on the provision of health services | Affected by the crisis, participants shared their feelings, and emotions. They discussed the adverse effects of the crisis on health care in Lebanon and the worsening biases in care due to the crisis. |
